# ‘Our courage has grown’: a grounded theory study of enablers and barriers to community action to address violence against women in urban India

**DOI:** 10.1136/bmjgh-2022-011304

**Published:** 2023-01-23

**Authors:** Lu Gram, Sukanya Paradkar, David Osrin, Nayreen Daruwalla, Beniamino Cislaghi

**Affiliations:** 1 Faculty of Population Health Sciences, Institute for Global Health, University College London, London, UK; 2 Society for Nutrition, Education and Health Action (SNEHA), Mumbai, India; 3 Department of Global Health and Development, London School of Hygiene & Tropical Medicine, London, UK

**Keywords:** Injury, Prevention strategies, Qualitative study, Mental Health & Psychiatry

## Abstract

Transforming communities into supportive environments for women facing risks of violence requires community members to play an active role in addressing violence against women (VAW). We did a grounded theory study of enablers and barriers to community response to ongoing violence, sampling from programme areas of a non-governmental organisation (NGO)-led community mobilisation intervention in informal settlements in Mumbai, India. We held 27 focus group discussions and 31 semistructured interviews with 113 community members and 9 NGO staff, along with over 170 hours of field observation. We found that residents responded to violence in diverse ways, ranging from suicide prevention to couple mediation to police and NGO referral. Enabling and constraining factors fit into a social ecological model containing intrapersonal, immediate social network, and wider societal levels. We identified four themes interlinking factors: legitimacy of action, collective power, protection against risk and informal leadership. Legitimacy of action was negotiated in the context of individual disputes, making community members question not only whether VAW was ‘wrong’, but *who* was ‘wrong’ in specific disputes. Collective power through neighbourhood solidarity was key to action but could be curtailed by violent gang crime. Interveners in incidents of VAW turned out to need significant physical, social and legal protection against reprisal. However, repeat interveners could become informal leaders wielding influential prosocial reputations that incentivised and facilitated action. Our model integrates multiple perspectives on community action into one analytical framework, which can be used by implementers to ensure that community members receive encouragement, support and protection to act.

**WHAT IS ALREADY KNOWN ON THIS TOPIC**

- Community mobilisation interventions can create enabling environments for community action, but results have proved variable.

**WHAT THIS STUDY ADDS**

- We present a novel social ecological model identifying factors promoting or hindering community action in response to violence against women.
- Four key themes were identified: legitimacy of action, collective power, protection against risk and informal leadership.

**HOW THIS STUDY MIGHT AFFECT RESEARCH, PRACTICE OR POLICY**

- Community members play a key role in addressing violence against women, but often need encouragement, support and protection to do so.

## Introduction

Transforming communities into safe and supportive environments for women facing risk of violence has long been a goal for feminist activists and researchers.^1^ Communities may play an unhelpful role by upholding social norms that blame survivors for experiencing violence, absolve perpetrators of accountability and ostracise women who speak out.^2 3^ Alternatively, communities may play a positive role by raising awareness, providing social support for survivors and intervening in cases of violence.^1^ Mobilising communities to act in the face of pervasive violence requires an understanding of community action. We propose a novel conceptual model of enablers and barriers to such action based on a study of informal settlements in Mumbai, India.

### Background

Violence against women (VAW) is recognised as a profound human rights and public health concern, with severe human, emotional and economic costs.^4^ Male intimate partners are the most common perpetrators and 27% of women globally are estimated to have experienced physical or sexual intimate partner violence in their lifetime.^5^ International declarations such as the United Nations Sustainable Development Goals have committed national governments to eliminating VAW and promoting women’s empowerment.^6^ Alongside widespread calls to action, the evidence base on what works to prevent VAW is evolving.

Interventions that mobilise communities to tackle social and structural drivers of VAW present some of the most effective known examples.^7^ They can be defined as interventions in which communities collaborate with implementing organisations in identifying, prioritising and tackling causes of ill health based on principles of bottom-up leadership and empowerment.^8^ Interventions in Uganda have reduced rates of violence by training volunteer activists to diffuse anti-VAW messages, take action against domestic violence and organise campaigns and marches.^9 10^ Implementers who worked with communities to change social norms and promote community action broadly theorised that collective power to create social change arises after a period of awareness raising.^9^

However, evaluations of interventions in Rwanda, Ethiopia, South Africa, India and Nepal have failed to show similar effects, raising questions about how intervention processes and impacts vary across contexts.^11–15^ Theories of community-based intervention have been criticised for exhibiting ‘positive *a priori* bias’,^16^ in which community engagement is assumed to elicit a positive response without complication. Profound challenges to participation in grass-roots collective action often coexist with enthusiasm for community engagement.^15 17 18^ These include people’s struggle for survival in conditions of poverty, lack of time to engage with the topic and poor trust among community members.

To address this evidence gap, we did a study of enablers and barriers to community action in Mumbai, India. We sampled from programme areas of a non-governmental organisation (NGO)-led community mobilisation intervention in informal settlements.^19^ We focused on community responses to incidents of VAW and show that residents responded in diverse ways spanning emergency response, survivor and perpetrator engagement and institutional referral. We also show that a multiplicity of factors governed the emergence of community action—some enabling, some hindering—and present a comprehensive organising framework.

### Theories of community action

At the most general level, community action can be seen as a manifestation of the distributed agency of local residents.^20^ VAW is a collective issue for communities and is driven by individual and structural factors requiring action by multiple stakeholders.^21^ Women, men and survivors of violence experience interlinked vulnerabilities through shared social and gender norms, lack of institutional resources and poor material conditions.^22^ Communities often work together to address VAW as a shared goal rather than as a collection of individuals with independent motivations.^23^ Individuals taking action against VAW can be seen as explicitly or implicitly taking part in collective action.

A number of theories potentially explain the (non-)emergence of community action. Political psychologists have argued that perceived legitimacy of action, perceived efficacy of action and social identity are important determinants of feminist collective action.^24^ Critical consciousness, visibility of VAW as a public issue, capacity for informal social control and community trust have been theorised as drivers of social change and action to prevent domestic violence in Northern India.^25^ In Peru and Rwanda, material and social incentives have mattered, as volunteer activists felt inadequately paid, but faced criticism of their authenticity when they were perceived to be compensated for their work.^26^ Social ecological models of bystander action to prevent sexual violence have been applied to US community and university campus settings,^27^ but not Global South contexts.

## Methods

Grounded theory is commonly used to inductively develop novel theories of understudied phenomena using qualitative data. We used Corbin and Strauss’ version^28^ to develop and extend theories of community action to address VAW for our context. We modified our sampling frame and topic guides over the course of data collection to explore new concepts. We followed an iterative loop of conducting focus group discussions (FGDs) and semistructured interviews (SSIs), doing observational fieldwork, transcribing and analysing data and sampling new respondents based on prior data analysis. We aimed to create in this process a rich, nuanced theory with a coherent, internally connected logic.

### Research context

Informal settlements are characterised by overcrowding, insubstantial housing, insufficient water and sanitation, insecure land and housing tenure and hazardous location.^29^ Most recent estimates from Mumbai find that 13% of women have survived physical violence, 4% sexual violence and 19% emotional violence in these settings in the last year.^30^ Women disproportionately bear burdens as primary caregivers for children and seniors and transgression of social and gender norms can lead to domestic violence.^31^ The stigma of divorce provides a strong disincentive for survivors to leave abusive marriages.^32^ Some communities also restrict women and girls’ mobility, dress and social interaction outside the home, creating public spaces that are visibly dominated by men and boys.^33^ Sexual harassment is common and often perpetrated by criminal youth gangs.^34^

In this context, the NGO Society for Nutrition, Education and Health Action (SNEHA) is implementing a community-based violence prevention programme delivering community mobilisation, crisis counselling and work with institutional services.^19^ Community mobilisation takes place through meetings of groups of women, men and adolescents who discuss community and gender issues with a facilitator and take action to address VAW. Female volunteers called *sangini*s (female friends) are trained to identify and support survivors through crisis intervention and referral to counselling, police and medical services. Male volunteers called *mitras* (male friends) are given training in male allyship with an emphasis on supporting *sanginis*. Counsellors provide immediate and longer term support to survivors, including mental health support and referral to mental health professionals, and link survivors to medical, legal, shelter and police services.^35^

### Sampling and recruitment

In line with theoretical sampling, we sampled communities, groups and individuals based on theoretically relevant categories in ongoing data analysis.^28^ First, we sampled respondents based on exposure to the community mobilisation programme using three categories: (1) general community members who had little to no exposure, (2) current members of NGO-run groups and (3) community volunteers who have received additional training and support. Within each category, we sought a degree of variation in respondent occupation, education and religion as these factors might affect participation in community action.^36^ We also worked with the NGO team to identify NGO-run groups who had been particularly active or inactive, so that we could directly ask about reasons behind participant (in)activity. We also sampled individuals from FGDs for follow-up interviews if they had been particularly vocal or silent, as they might face particularly strong enablers or barriers to community action. We sampled communities for additional FGDs and SSIs when data analysis revealed concepts in need of refinement, such as the role of neighbourhood cohesion in influencing willingness to act against VAW. Apart from these considerations, any community member aged 18 or above was eligible to participate. NGO staff physically approached community members or called them on the phone, if they were registered with an NGO group, for recruitment. We also sampled NGO staff for focus groups and interviews to explore their perspectives. Finally, we did unstructured, non-participant observational fieldwork to learn about communities in ways not possible in a formal interview setting.

In total, we conducted 16 FGDs with 75 women, 6 FGDs with 31 men, 19 SSIs with women and 8 SSIs with men. Amongst these, we included repeat FGDs with three groups of women and repeat SSIs with three women who presented particularly rich data in need of further elaboration. We also included interviews with one male and one female resident who had stopped participating in NGO activities to triangulate the reports of people who were still involved with the reports of people who had left. Table 1 lists the characteristics of FGDs and table 2 the SSIs with community members. In addition, we conducted five FGDs and four SSIs with NGO staff, including counsellors, community organisers, programme officers and a programme coordinator. In total, we spoke to 113 community members and nine NGO staff.

**Table 1:** List of focus group discussions with community members

ID	Sex	Type	Expected activity level	Participants (n)	NGO exposure
FG1	Female	General	N/A*	5	None
FG2	Female	General	N/A*	7	None
FG3	Female	Group members	Active	6	4 years
FG4	Female	Group members	Inactive	6	3–5 years
FG5	Female	Group members	Active	5	3 years
FG6	Female	Group members	Inactive	5	3 years
FG7	Female	Group members	Inactive	6	2–4 years
FG8	Female†	Group members	Active	8	3 years
FG9	Female	Volunteers	Active	7	1–15 years
FG10	Female	Volunteers	Active	5	8–14 years
FG11	Female	Volunteers	Inactive	4	3–7 years
FG12	Female†	Volunteers	Inactive	5	3 years
FG13	Female†	Volunteers	Active	6	1–6 years
MG14	Male	General	N/A*	5	None
MG15	Male	General	N/A*	5	None
MG16	Male	Group members	Active	4	4 years
MG17	Male	Group members	Inactive	7	1.5 years
MG18	Male	Volunteers	Active	5	2–13 years
MG19	Male	Volunteers	Inactive	5	1.5–4 years

‘Volunteers’ are NGO-trained community volunteers. ‘Group members’ participate in NGO-run group meetings in the community. ‘General’ refers to general community members who are neither volunteers nor group members. ‘Expected activity level’ refers to the degree to which NGO staff judged the group to be actively engaged in tackling VAW—rather than passive—at the stage of sampling.

*NGO staff cannot judge activity levels of community members who do not join NGO events.

†Received a follow-up focus group discussion.

FG, focus group; NGO, non-governmental organisation; VAW, violence against women.

**Table 2:** List of semistructured interviews with community members

ID	Sex	Type	Age (years)	Marital status	Religion	Education	Occupation	Interview mode
FI1	Female	General	26–35	Married	Muslim	5th grade	Home maker	Face to face
FI2	Female	General	56–65	Widowed	Muslim	12th grade	Home maker	Face to face
FI3	Female	General	56–65	Married	Muslim	<5th grade	Stitching work	Face to face
FI4	Female	General	26–35	Married	Hindu	4th grade	Home maker	Face to face
FI5	Female	Group member	36–45	Married	Muslim	None	Home maker	Face to face
FI6	Female	Group member	36–45	Married	Hindu	8th grade	Electrical parts	Face to face
FI7	Female	Group member	36–45	Married	Hindu	10th grade	Home maker	Face to face
FI8	Female	Group member	36–45	Married	Muslim	<5th grade	Home maker	Face to face
FI9	Female	Group member	36–45	Married	Hindu	12th grade	Teacher	Face to face
FI10*	Female	Group member	36–45	Married	Muslim	<5th grade	Home maker	Face to face
FI11	Female	Volunteer	36–45	Married	Muslim	8th grade	Home maker	Face to face
FI12	Female	Volunteer	36–45	Married	Hindu	10th grade	Beauty parlour	Face to face
FI13	Female	Volunteer	36–45	Married	Muslim	8th grade	Home maker	Face to face
FI14*	Female	Volunteer	46–55	Married	Hindu	7th grade	Shop owner	Online
FI15	Female	Volunteer	26–35	Married	Muslim	5th grade	Stitching work	Online
FI16	Female	Volunteer	36–45	Married	Muslim	7th grade	Home maker	Face to face
FI17*	Female	Volunteer	36–45	Married	Hindu	8th grade	Stitching work	Face to face
FI18	Female	Volunteer	36–45	Married	Muslim	<5th grade	Home maker	Face to face
FI19	Female	Ex-volunteer	20–25	Married	Muslim	9th grade	Home maker	Face to face
MI20	Male	General	20–25	Unmarried	Hindu	12th grade	Sales	Online
MI21	Male	General	36–45	Married	Muslim	5th grade	Welding	Online
MI22	Male	Group member	20–25	Unmarried	Hindu	Bachelor’s	Banking	Online
MI23	Male	Group member	26–35	Married	Muslim	8th grade	Transport	Online
MI24	Male	Volunteer	46–55	Married	Hindu	10th grade	Driver	Face to face
MI25	Male	Volunteer	20–25	Unmarried	Buddhist	Bachelor’s	Municipal worker	Face to face
MI26	Male	Volunteer	26–35	Unmarried	Hindu	Bachelor’s	Unemployed	Online
MI27	Male	Ex-volunteer	56–65	Married	Buddhist	9th grade	Garments	Online

‘Volunteers’ are NGO-trained community volunteers. ‘Group members’ participate in NGO-run group meetings in the community. ‘General’ refers to general community members who are neither volunteers nor group members.

*Received a follow-up interview.

NGO, non-governmental organisation.

### Data collection

Data collection took place in 2021–2022 in Dharavi and Govandi, two large informal settlements in Mumbai, India. Author SP conducted all FGDs and SSIs, which were in Hindi or Marathi and lasted between 30 and 90 min. They were conducted online over Google Hangouts or face to face and were recorded. Participants in face-to-face sessions were asked to watch video clips, make drawings or act out short scenes as prompts for discussion. The video clips presented short (2 min) scenes of couple fights escalating into violence (shouts, name calling and a slap). The drawing exercises asked participants to diagram people close to them and explain why they mattered to them. In the acting exercises, participants enacted past experiences of being approached for help by survivors. SP and two translators transcribed 10% of recordings into Hindi or Marathi first and then translated them into English. Author LG listened to audio recordings and read through transcripts in Hindi and English to check translation quality and discuss interview techniques with SP. SP checked the Marathi segments by the two additional translators. The remaining recordings were translated directly into English.

SP conducted over 170 hours of observational fieldwork through repeated visits to communities to observe meetings with community members, NGO staff ‘doing rounds’—going from house to house and speaking to community members—as well as awareness-raising campaigns. SP spent time ‘hanging out’ in the community, engaging in informal conversations with women and observing interactions between them on the street or in their homes. SP conducted observations in an open-ended manner, following a template directing them to pay attention to the physical environment, local people and their conversations. Field notes were recorded on paper and transcribed digitally. LG held debriefing sessions with SP after each FGD, SSI and field visit to discuss new concepts and plan avenues of enquiry. Both continually revised topic guides in light of prior data analysis, resulting in unique topic guides for each FGD and SSI.

Overall, topic guides focused on eliciting stories of community (in)action and exploring enablers and barriers to action. We asked about community members’ experiences engaging with NGOs, how they reacted to incidents of VAW, what motivated them to take action, how others viewed participation in such action and how they decided what action to take. We also asked about residents’ views on VAW, whether intervening in incidents of VAW was justified and whether they felt a need to change local attitudes and social norms. Topic guides for NGO staff asked about the role of communities in the VAW prevention strategy and their experiences in engaging community members.

### Data analysis

LG and SP coded transcripts of FGDs, SSIs and field notes using the software tool MAXQDA 2020. For each transcript, reflective notes were written using analytical tools from grounded theory, such as ‘waving the red flag’^28^ whenever transcripts involved the use of words such as ‘always’ or ‘never’ to question researcher assumptions. This was followed by open coding, in which the text was broken up into discrete chunks and labelled, and then axial coding, in which different codes were connected and grouped into categories. We applied constant comparison and compared codes within a single interview and between interviews. In particular, we assiduously compared individuals, groups and communities showing greater or lesser activity levels in addressing VAW.

At the selective coding stage, we arranged categories relating to enablers and barriers to community action using social ecological levels.^37^ As individual factors did not act in isolation, but interacted, we additionally grouped codes into cross-cutting themes to show linkages between social ecological levels. This study was not an intervention evaluation, but aimed to take a whole-system approach to community action, so we aggregated findings for general community members, NGO-run group members and community volunteers.

To reduce cultural bias, stimulate reflective analysis and ensure analytical rigour, LG and SP discussed findings and interpretations with each other, coauthors and NGO staff throughout data collection and analysis on a near-daily basis. This enabled us to consider surprising findings, question assumptions and critically examine our own role in generating theory from data. SP phoned past interviewees to clarify unclear statements. At the end of data analysis, a meeting was held with NGO staff to discuss findings and receive feedback.

### Patient and public involvement

NGO staff were involved throughout in discussing interpretations of data and providing feedback to our analysis. Community members were not involved in the design, conduct, reporting or dissemination plans of this research. An author reflexivity statement is provided in online supplemental appendix S1.

10.1136/bmjgh-2022-011304.supp1 Supplementary data

### Ethics

We followed the WHO guidelines for research on domestic VAW.^38^ We provided participant information sheets to respondents, discussed the nature of the interview and obtained consent. For face-to-face interviews, we obtained signed consent. When participants could not read or write, we took thumb prints. For online interviews, we took verbal consent. Recordings were stored securely and wiped after transcription with all names and identifiers replaced by pseudonyms. We also obtained informed consent for field observations from NGO staff and community members. Where observation yielded personally sensitive information, we double-checked with staff and community members that they agreed to us recording it in our field notes in anonymised form. A protocol was followed for action in cases of disclosure of abuse or signs of distress from participants, including referral to available services for survivors of violence.

## Results

In the following sections, we will first describe community responses to VAW and then present our conceptual model of enablers and barriers to community action.

### Community responses to VAW

Table 3 lists the community actions reported by participants in response to incidents of VAW.

**Table 3:** Community actions in response to violence against women

Type of action	Examples
**Emergency intervention**	
Providing crisis support to survivors	Intervening in suicide attempts by survivors
	Taking survivors to hospital for injuries
	Extracting survivors from abusive homes
Interrupting ongoing violence	Physically separating couples or stopping violence
	Shouting at or distracting perpetrators
	Calming down couples or perpetrators during fights
**Survivor support**	
Providing informal support to survivors	Encouraging survivors to resist violence
	Providing emotional support to survivors
	Donating money or items to survivors
Promoting couple communication	Encouraging reconciliation and mutual forbearance
	Mediating or arbitrating in family disputes
**Perpetrator engagement**	
Verbally sanctioning perpetrators	Admonishing perpetrators for doing wrong
	Confronting perpetrators over their actions
	Showing perpetrators that they are being unreasonable
Threatening or punishing perpetrators	Threatening police action or imprisonment
	Threatening case registration with NGO
	Threatening or effecting violence against perpetrators
**Institutional referral**	
Connecting survivors to NGO services	Providing contact details of NGO to survivors
	Notifying NGO staff of new cases of violence
	Getting survivors to visit and register with NGO
Getting the police to respond to cases of violence	Asking police officers to speak to perpetrators
	Helping survivors file cases against perpetrators
	Helping police catch and prosecute perpetrators

NGO, non-governmental organisation.

#### Emergency intervention

Neighbours were often the only people physically available in emergencies. Community members had prevented suicide attempts by survivors, taking injured women to hospital in the middle of the night, or extracted them from abusive homes into places of shelter. Many had interrupted violent perpetrators in the act by physically preventing them from striking a woman, distracting them or persuading them to calm down. For example, a member of an NGO-run men’s group stopped his drunk neighbour from further violence by asking him to go to sleep:

When we came to know what was going on, we immediately went and separated husband and wife… We calmed them down, talked to them. He was drunk at the time. So, we asked him to sleep, ‘We’ll talk tomorrow morning, ok?’ We talked him over and got him to sleep. (Interview with male group member, MI22)

#### Survivor support

Beyond specific incidents, community members had often provided informal support to survivors, for example, by providing emotional support, advice or encouragement for women to tell their stories, seek help or resist giving in to abusers by accepting violence inflicted on them as part of their lot. They heard out survivors’ painful experiences and reassured them of being there for them. Survivors experiencing acute poverty due to neglect or abandonment were at times offered money or items. In cases of household conflict, community members encouraged fighting parties to show tolerance and mutual forbearance, sometimes playing the role of mediator or arbitrator. For example, one female volunteer had convened a joint meeting of 10–15 people from a woman’s marital and natal homes after the woman had tried to poison herself. Through dialogue and diplomacy, she got the husband to agree to stop the abuse. She explained her process as follows:

I neither let anyone gain unfair advantages nor let anyone lose out. Whatever’s troubling her, I let her speak about it. It’s not like, I ask one person to speak, and another to remain silent. I say helpful things to everybody. Even if there are nasty things which someone has done or said [without the other person’s knowledge], I don’t disclose it to the others. Why? Because she also needs to move on. One shouldn’t drag a woman down but encourage her to grow. (FGD with female volunteers, FG9)

#### Perpetrator engagement

Participants had frequently engaged perpetrators by admonishing or arguing with them to show that they were being unreasonable. For example, a male volunteer had sought to persuade a friend that punishing his wife for ‘failing’ to birth a son was illogical, ‘*You are a farmer, so you sow seeds in the field… Whatever seed goes into the body determines whether you will get a boy or a girl*’ (FGD with male volunteers, MG18). A female group member had argued with a man who was sexually harassing girls, telling him that he was damaging their children’s education, ‘*We don’t have any other road we can travel through! If our children get scared off by this, they might even stop going to tuition and school*’ (FGD with female group members, FG8).

At the more combative end of interventions, participants had threatened perpetrators with police action, jail or case registration with NGOs dealing with VAW. In certain cases, community members had even threatened or carried out violence against perpetrators.

If a husband is beating his wife, then–from what I have seen–people in the neighbourhood intervene. They force the husband aside, sometimes even hit him, and ask him, ‘Why are you beating your wife?’ They beat him up or try to talk to him. If after all that, he still doesn’t get it, then they just tie him up and call the police. (Interview with male community member, MI20)

#### Institutional referral

Residents had also engaged local institutions. Some had left the contact details of relevant NGOs with survivors. Most had made significant efforts to ensure that survivors met with a case worker and registered their case, for example, by accompanying them to the NGO office. Survivors were often frightened and reluctant to talk to NGO workers for fear of escalating their family situation. For the same reason, residents often accompanied survivors to the police station to file a report against perpetrators. In severe incidents involving rape or femicide, participants had helped the police to apprehend perpetrators and collect evidence for prosecution. For example, one volunteer had asked around in the neighbourhood together with three friends for the whereabouts of a serial child molester who had abused her own son and multiple girl children. When they found his house, they talked to his parents to confirm his identity and then physically guided the police there to arrest him (Interview with female volunteer, FI11).

### Social ecological model

**Figure 1:**
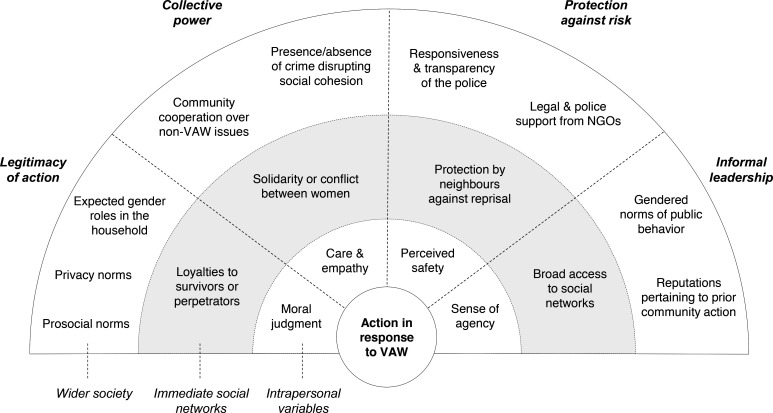
Factors shaping action in response to violence against women (VAW). Factors act as enablers or barriers depending on their instantiation. For example, solidarity between women promotes action, whereas conflict hinders it. NGO, non-governmental organisation.

Figure 1 presents our conceptual model of community action. The model has factors shaping action at intrapersonal, immediate social network, and wider societal levels. Four cross-cutting themes connect factors across levels: (1) ‘legitimacy of action’ or the extent to which action to address VAW was socially constructed as legitimate, (2) ‘collective power’ or the capacity to draw on relationships of trust and solidarity to act, (3) ‘protection against risk’ or the degree to which community members were protected from possible reprisal for action and (4) ‘informal leadership’ or the extent to which initiators of action became informal leaders.

#### Legitimacy of action

##### Intrapersonal variables

The degree to which individuals saw action to address VAW as morally justified played a role in motivating or discouraging action. This was starkly exemplified by most respondents—even those vocally speaking out against VAW—expressing a need to assess who among disputing parties was ‘right’ or ‘wrong’ before intervening in violent incidents. For instance, a female NGO group member explained that, in case of domestic violence, a survivor’s father would first ask the perpetrator’s family what she had done wrong:

Respondent: First, they’ll talk it over with the husband’s family members and ask them, ‘Why are you doing this to her? What has my daughter done wrong?’ … Only then can one know what one should do – if we should reason with the girl or say something to the boy.

Interviewer: Ok. If they can’t resolve the issue even after talking to the in-laws, what then?

Respondent: They will tell you about the girl’s mistakes. After which the father says, ‘Yes, my daughter made a mistake, so I can’t do anything about this.’ (Interview with female group member, FI6)

Only if it was known that she had made ‘no mistakes’ could he intervene. Conversely, perpetrators clearly seen to be wrong elicited strong emotions of anger, outrage or disgust motivating action, ‘*I cannot tolerate [sexual harassment of girls], my blood starts boiling… I am responsible for my daughter’s safety*’ (Interview with female group member, FI5).

##### Immediate social networks

Existing loyalties between community members, survivors and perpetrators influenced who was seen as ‘wrong’ and therefore whether action was seen as legitimate. Mothers and male friends of perpetrators were liable to believe perpetrators’ accounts and dismiss survivors’ accounts as exaggerations or false claims. A male volunteer explained how he interpreted physical injuries suffered by his friend’s wife as self-inflicted. It was unclear if he had spoken to the wife at all, but he believed his friend:

She was hitting her own hand, drinking phenyl [a chemical used to attempt suicide] … she was doing it in the middle of the night and so my friend suffered. She went out at night and told others in the community that he was forcing her to drink phenyl and abusing her. In fact, he’s never done anything like that, and he was so nice that he shared all this with me! (FGD with male volunteers, MG19)

NGO staff were well aware of the potential for existing loyalties to bias judgement. One cited a past project to establish a community-run federation to address VAW, which had included a ‘ground rule’ forbidding members from leveraging their influence to protect relatives who were perpetrators of violence (Interview with NGO staff).

##### Wider society

Multiple, intermingled norms delegitimised action to address VAW. Determining the extent to which perpetrators or survivors had been ‘wrong’ in a domestic dispute involved invoking gendered expectations of male authority and women’s fulfilment of domestic roles. As reported in the past,^18^ inaction in the face of domestic violence was often justified with reference to norms framing it as a private matter. Residents approved of a broader norm of ‘minding your own business’, which was contrasted favourably with noisily fighting with neighbours or snooping on others’ affairs, often seen as typical of life in the rural village. In a few places, new tower blocks built by the Maharashtra Housing and Development Authority to provide affordable housing for local residents even allowed for a high degree of noise insulation (Ethnographic field notes).

However, set against these norms were norms of prosociality and altruism. ‘Helping each other out’ was seen as necessary to prepare for future emergencies by building networks of mutual aid with neighbours, to earn good *karma* for future lives and to avoid divine ill judgement. In specific incidents of violence, multiple norms were active at once. One male group member explained how neighbours tried to discourage him from intervening in an incident of domestic violence by saying, ‘*He’s a drunkard! This is what they do!*’ or ‘*Why are you intruding?*’ He eventually intervened because ‘*If we don’t stand up for others today, who will stand up for us tomorrow?*’ (Interview with male group member, MI22).

#### Collective power

##### Intrapersonal variables

Care and empathy for women in the community, including survivors of violence, formed a foundation for neighbourhood solidarity. Active respondents described acting out of an overpowering urge to help when faced with the scale of other women’s suffering. They recalled details such as survivors ‘almost going crazy’, ‘bleeding from the mouth’, crying or experiencing mental health problems, which often went unnoticed in accounts by less active respondents. Some related survivors’ experiences to their own, ‘*I have been through this, so I don’t want another woman to go through the same*’ (Interview with female volunteer, FI15). Others experienced strong emotions simply narrating events:

I felt like crying because that woman had suffered so much abuse and for what! I mean, her parents must have taken great care of her, the way you take care of a flower, and now after marriage, she has to face so much abuse. I mean her hair… and the chilli powder… [put into her private parts during intercourse] And he’d abuse her so much! I mean, I think he was mentally unstable–whether mentally ill or psychotic, they must have figured out later–but I felt awful and I felt like crying when I… Then I spoke to [NGO] staff about it and afterwards, we got her admitted to hospital. (Interview with female group member, FI9)

##### Immediate social networks

Female solidarity was key, as action to address VAW often required a physical group to be effective. Persuading perpetrators and their families to listen was easier in a group, stopping physical fights often required multiple interveners and police and government officers were less liable to dismiss the concerns of survivors when faced by a group. For example, one respondent expressed a sense of confidence in collective intervention in cases of VAW by saying, ‘*When we are together then we can solve any problem. When ten women get together then one man can’t do anything*’ (FGD with female group members, FG5). A perceived lack of solidarity could undermine motivation to act. One volunteer felt so let down by her fellow women’s group members, when a local gang threatened her business, that she stopped participating in NGO activities and nearly refused an interview with us (Interview with female volunteer, FI12).

##### Wider society

Neighbourhood cooperation over issues not directly related to VAW supported solidarity in the face of VAW. Basic neighbourhood amenities such as water and electricity, garbage collection and waste disposal, or street repair and cleaning were often lacking or unreliably supplied. NGO staff facilitated petitions, protests or collective self-help to improve these amenities as part of an intentional strategy to demonstrate the value of collective organising:

If the shopkeeper in charge refuses to give them their rations or what they get is of poor quality… [or] the garbage collector doesn’t show up for work, and the garbage piles up, or sometimes sewers overflow, etc. They can’t deal with all these things alone. It’s much better if they do it in a group. That’s how women end up thinking, ‘Yes, we need to have a group!’ (Interview with NGO staff)

This could be challenging to organise in some communities with pervasive crime, as residents cited graphic examples—backed up by video and photograph evidence circulating on social media—of violent punishment for getting on the wrong side of local criminals. For example, one man had been stabbed for refusing to ‘donate’ his phone to two gang members passing by. When neighbours tried to intervene, they were also killed. In the same community, women feared punishment for their husbands or sons if they spoke up, citing graphic punishments involving fingers being cut-off with swords in the past (Ethnographic field notes).

#### Protection against risk

##### Intrapersonal variables

Perceived safety of intervening shaped willingness to act. Residents often spoke about community action requiring *himmat*, which translates to ‘courage’ or ‘daring’, and with good reason. One community member was hit by a stone when she tried to speak to a perpetrator. Another was beaten by the perpetrator when she tried to physically separate a fighting couple. Still others had faced police or legal challenges from perpetrators’ families accusing them of trespassing or violating privacy laws in retaliation for intervening. As one volunteer put it, when you try to intervene in a domestic dispute, often ‘*your dispute starts after their fight ends*’ (Interview with male volunteer, MI25). Respondents who rarely participated in VAW prevention activities spoke about their fear of reprisal, while respondents who were regularly active underscored their resilient nature with statements such as, ‘*One thing about me: I am not scared of fisticuffs*’ (Interview with female volunteer, FI14).

##### Immediate social networks

Protection from neighbours partially mitigated risks of action. Showing up in a physical group was more intimidating than a lone intervener and could discourage perpetrators from retaliating. ‘Difficult cases’ could be delegated to well-connected community members whom perpetrators would be more hesitant to cross, ‘*Whenever there’s a fight, she’s first to intervene – not me. Because I’m a bit afraid, but she’s cool and carefree… I tell her to speak to them because she knows a lot of people*’ (FGD with female volunteers, FG11). Legal risks were reduced when intervening in a group, as other participants could serve as witnesses in case of a legal challenge (Interview with NGO staff). One volunteer cited common a South Asian metaphor that ‘*Women are like sticks. When they all come together, they are stronger*’ (Interview with female volunteer, FI11).

##### Wider society

Support from NGOs such as SNEHA constituted powerful motivators, as protection from neighbours alone was rarely thought sufficient. Amidst widespread reports by community members of unresponsiveness and corruption among the police, NGOs were seen as having a greater degree of clout than ‘common people’ by virtue of their high status in society and knowledge of legal procedures, ‘*No-one gives a hearing to common people. Unless a higher-up person comes along, no one gives a hearing… Someone with a big name needs to come along*’ (FGD with female volunteers, FG12). NGO counsellors who were trained social workers, but not legal professionals, were sometimes referred to as ‘lawyers’, ‘*I’ve spoken to a personal lawyer as well, the big female lawyer, the lady who visited us! She was the biggest lawyer of them all*’ (FGD with female group members, FG4). Although NGO support did not universally inspire confidence—for example, in communities with entrenched violent gang crime—many respondents felt that their *himmat* or ‘courage’ had grown as a result:

We’re no longer afraid, our courage has grown. Since we joined SNEHA, I haven’t had tension about anything. I’ve stopped being afraid and I now act fearlessly without hesitation. Earlier we used to be afraid of intervening anywhere, like if there was any kind of fight or tension anywhere, now we move around fearlessly, we can come and go anywhere we want! (FGD with female group members, FG5)

#### Informal leadership

##### Intrapersonal variables

Informal leadership for many female respondents often began with greater sense of agency from joining NGOs. Previously, these women had rarely left their home or neighbourhood except to visit their natal village. The experience of joining a social network distinct from their family, gaining access to the ‘world outside’ their home and learning about themselves as rights bearers in Indian law felt like a transformation from a sheltered, ‘gullible’ housewife to a savvy, outgoing woman capable of navigating police and court processes. This inspired them to support others and resist violence in their own lives.

I started saying to him, ‘No, you can’t beat me! This is not what a husband and wife should do! We need to be there for each other… I help others and if you come across someone in need, you can also help them! If you think that I’ve done something that you don’t like, then you can tell me, but you can’t raise your hand and hit a woman like this!’ Then my husband said, ‘Where did you learn this? A woman like you who spends all day at home…’ I said, ‘Women usually don’t get out of the house, but as soon as they do, they learn to fly.’ (Interview with female group member, FI7)

##### Immediate social networks

Broad access to social networks was necessary for the development of informal leadership. Women who reported little action to address VAW sometimes explained that continuing family restrictions on their mobility prevented them from building the close relationships required to learn about violence happening to other women. At the same time, male social networks were overwhelmingly homosocial: ‘*We’ve nothing to do with the women here in Mumbai. Even if I knew someone, she wouldn’t tell me anything about her personal life. Outside the family, there’s neither a need for us to ask questions of women nor a chance that they might tell us anything*’ (FGD with male community members, MG15). This made it difficult to identify cases of violence, and increased the likelihood that men only heard the perpetrator’s version of events when violence did happen.

##### Wider society

Gendered norms of public behaviour shaped men and women’s access to social networks. Men were mostly friends with other men due to taboos on socialisation between men and ‘non-kin women’, or *gair-mahila* in Hindi, rendered cross-gender friendships fraught with risk of accusations of (sexually) improper behaviour. For example, a male neighbour of a female volunteer refused food from her despite starving for fear of rumours (FGD with female volunteers, FG10). Family restrictions on women’s mobility followed normative templates for ‘acceptable’ reasons for women being in public, such as collecting rations for the household, not socialising, making friends or ‘wasting time’. In some communities, women even feared breaking these norms in secret, as neighbours might tell their family, ‘*The men… They come to know everything*’ (Interview with female group member, FI10).

However, residents who did succeed in taking community action to address VAW accumulated name recognition, esteem and influence over time, which provided incentives for them to continue their work. They developed reputations for goodness, helpfulness and bravery, and became go-to persons for issues of VAW. This significantly facilitated support for survivors of violence as cases of VAW were obviously easier to identify when survivors themselves came forward asking for help. One volunteer became a leader of her own women’s group and nailed a wooden board to the front door of her house advertising free services for survivors of violence (Ethnographic field notes). She became widely known for her activism and even received requests for support from outside her informal settlement. Community members said that people saw her as a role model and were asking her to stand in elections: ‘*She’s established this image of herself from the very beginning, and that’s why people listen to her … Everyone really respects her*’ (Interview with male community member, MI20).

## Discussion

We built an empirically grounded model identifying factors promoting or hindering community action in response to VAW in informal settlements in Mumbai, India. Rather than favouring any single explanation, our model spanned multiple theoretical paradigms. Our theme of legitimacy of action concurred with theories of perceived legitimacy and critical consciousness.^24 25^ Our theme of collective power aligned with theories of social identity and empowerment.^24 25^ Impacts of social rewards through opportunities for leadership and costs through risks of retaliation on the decision to act were predicted by incentive-based theories.^39^ We integrated complementary explanations into a social ecological framework^27^ with factors at intrapersonal, immediate social network, and wider societal levels. Expanding scarce evidence on mechanisms of community action,^23^ our analytical framework provides structured guidance on key factors.

Our findings extend existing understandings of the role of gender and privacy norms in delegitimising action to address VAW.^17 25 26^ We found that general beliefs about the rightness or wrongness of VAW may play a smaller role in motivating action than specific beliefs about the ‘rightness’ or ‘wrongness’ of *people* involved in violent situations. Firm opposition to VAW in general could coexist with spirited defence of perpetrators when survivors were judged to be on the ‘wrong’ side of disputes. Such judgements were inflected by normative beliefs about gender roles, and epistemic beliefs on the veracity of claims by disputing parties. Indeed, past evidence from the Global North has found that perceived ambiguity over whether emergency situations are truly problematic can deter bystander intervention.^27^

Our theme of collective power highlights the well-documented role of solidarity and social capital in supporting community action.^25^ Consonant with reports from informal settlements in South Africa,^17^ we found that violent crime significantly damaged social cohesion. However, we also found that emotions of care and empathy—in addition to arousal^27^ and anger^24^—helped build solidarity. This is supported by quantitative evidence on the positive impacts of victim empathy on bystander action in Vietnam,^40^ and qualitative evidence on women’s care labour in networks of mutual aid in India.^41^ Combined with the finding that fear of backlash deterred action while feelings of courage promoted it, our results spotlight the underemphasised role of emotions in community action.

Community members taking action faced real risks of reprisal from perpetrators and their families. Past studies in the Global South have reported on social and financial costs—such as men facing criticism for transgressing gender norms or activists feeling inadequately remunerated–but not risks of reprisal.^17 25 26 42^ Differences in study aims may account for this, as we focused on enablers and barriers to community response rather than primary prevention. Financial compensation may loom larger as a consideration in decisions on participation in awareness-raising campaigns than in decisions on responding to ongoing violence. Our findings underline the urgency of calls to investigate how to encourage helpful behaviour while ensuring that this is done safely.^27^

Informal community leadership both incentivised and facilitated community action in our data. While participants in community mobilisation programmes are frequently labelled ‘change makers’ or ‘natural leaders’, this has often implied little more than extra training, confidence in public speaking and enthusiasm for activism.^43 44^ We contribute to evidence on more formal leaders such as faith leaders, committee members or village elders^23 45^ by showing that informal community members can become significant leaders—with attendant status and privileges—in their own right through repeated engagement with VAW prevention.

Not all actions taken by community members were unambiguously constructive. Some community members had engaged in threats of violence or carried out violence to punish perpetrators. These findings problematise predominantly positive conceptualisations of community action,^23^ and raise complex ethical questions about the extent to which such acts should be considered pure ‘vigilantism’, or legitimate responses to systemic failures to uphold women’s rights, or both.^46^ Rather than seek a definitive answer, it may be more important for researchers to interrogate the conditions under which community members come to view violent justice seeking as necessary in the first place.

### Limitations

Article space constraints prevented us from systematically exploring many topics, including gender differences in enablers and barriers to action, how community members decide on a course of action in response to VAW and what motivates participation in primary prevention activities rather than response to violence. In particular, it is important to note that our four themes—legitimacy of action, collective power, protection against risk and informal leadership—were mutually beneficial and had synergistic effects. Future papers will address these topics. Self-reports by study participants on their experiences with community action might have been coloured by desires to impress the interviewer. We mitigated this risk by triangulating against field observations, discussions with NGO staff who routinely log community actions and cases of VAW in a central database, and interviews with community members who had never been involved in the programme or had left it. Although new analytical insights became rarer towards the end of the study, indicating approaching saturation, some concepts remained unsaturated. Macro factors such as the functioning of Indian courts or mass media portrayals of gender may have affected community action, but could not be explored with our data.

### Implications for research and practice

Future researchers can use our framework to investigate community action to address VAW. Past research on women’s empowerment has conceptualised agency in individualistic terms using theories of intrahousehold bargaining power without measuring collective power.^47 48^ Our social ecological model can be used to extend measures of capacity for collective action to address VAW^49^ to better account for social network and wider societal drivers. Our model can also serve as an analytical framework for investigating mechanisms of future community mobilisation interventions in future process evaluations.^50^ This model was developed in the context of urban informal settlements in India; it might be fruitful to explore adaptations required for other contexts.

Collective action-oriented policy initiatives to address VAW are increasingly popular.^51 52^ Intervention designers may benefit from engaging with our themes, legitimacy of action, collective power, protection against risk and informal leadership. Consciousness-raising efforts could involve community members in critiquing specific incidents of violence, not only the general wrongness of violence. Neighbourhood solidarity and protection from backlash for interveners may require strengthening police and crime prevention units. Fostering informal leaders with a reputation for tackling VAW may help address well-known challenges in encouraging disclosure of VAW.^53^ Finally, community mobilisers should be aware of risks of vigilante violence and install safeguards for this eventuality.

## Data Availability

Data are available upon reasonable request. We informed participants as part of the consent process that we might share data with other researchers in anonymous form, but did not ask for consent to make the data available in the public domain.
