# Supplementary material for: ‘Our courage has grown’: a grounded theory study of enablers and barriers to community action to address violence against women in urban India
Source: BMJ Glob Health. 2023 Jan 23;8(1):e011304. doi: 10.1136/bmjgh-2022-011304 (PMC9872482; doi:10.1136/bmjgh-2022-011304)
Supplement: Supplementary data [file bmjgh-2022-011304supp001.pdf]

Appendix S1 – Reflexivity Statement

How does this study address local research and policy priorities?

1 in 4 women in India have experienced domestic violence in their lifetime. Community-based group interventions are increasingly popular tools to tackle poor health in India with significant policy interest in their application to tackling violence against women (VAW). Our study provides practical, actionable insights on how local policymakers and implementers can design more effective community-based interventions to address VAW.

How were local researchers involved in study design?

The study was originally conceptualised by the first author, a foreign researcher who has worked with South Asian partners for over a decade. The idea for the study grew out of prior collaborative work with local NGO partner SNEHA who was consulted at the design stage. As part of the grounded theory process, all decisions about sampling, analysis, and further sampling were taken jointly between the first author and the local researcher, so the local researcher had considerable influence over the direction of the study.

How has funding been used to support the local research team(s)?

With the exception of salary support for one foreign researcher and publication fees, the budget was spent entirely in India, on local staff.

How are research staff who conducted data collection acknowledged?

The sole data collector is also second author. Support by key people in the partner NGO in the data collection phase in e.g. finding and recruiting interviewees or organizing interviews in advance has been acknowledged in the Acknowledgements section of the paper.

How have members of the research partnership been provided with access to study data?

Data was collected by local staff who retained control. Original data was only available to SNEHA staff. Foreign researchers only had access to data in pseudonymized form.

How were data used to develop analytical skills within the partnership?

Data analysis was conducted jointly between the foreign researcher and the local researcher. NGO staff were regularly consulted in the interpretation of data.

How have research partners collaborated in interpreting study data?

We held regular meetings with research partners in interpreting the study data. At the end of data analysis, a meeting was held with NGO staff to discuss findings and receive feedback.

How were research partners supported to develop writing skills?

The foreign researcher encouraged the local researcher to draft sections of the manuscript and helped review and refine their writing.

How will research products be shared to address local needs?

The manuscript will be published as open access. The current findings form part of a larger research project involving qualitative, quantitative and experimental data. We intend to conduct dissemination meetings with local communities, SNEHA staff and managers, and interested policy stakeholders at the end of the project. We will also disseminate findings through media such as Twitter and LinkedIn.

How is the leadership, contribution and ownership of this work by LMIC researchers recognised within the authorship?

Two of the authors are Indian nationals based in India (SP and ND).

How have early career researchers across the partnership been included within the authorship team?

Early career researchers are part of the authorship team.

How has gender balance been addressed within the authorship?

3 authors are male (LG, DO, BC) and 2 are female (SP, ND).

How has the project contributed to training of LMIC researchers?

The second author is currently pursuing a PhD on women's activism in India.

How has the project contributed to improvements in local infrastructure

The project has not contributed to improvements in local infrastructure.

What safeguarding procedures were used to protect local study participants and researchers?

Near-daily debriefs were conducted between the foreign and local researcher, including debriefs on the mental well-being of the local researcher. In case of mental distress, a

psychologist was available from SNEHA. For details on the protection of local study participants, please see the Ethics section in the main manuscript.
